# Detailing the cardiovascular profile in shock patients

**DOI:** 10.1186/s13054-017-1908-6

**Published:** 2017-12-28

**Authors:** Daniel De Backer

**Affiliations:** Department of Intensive Care, CHIREC Hospitals, Université Libre de Bruxelles, Brussels, Belgium

## Abstract

Evaluation of the cardiovascular profile of critically ill patients is one of the most important actions performed in critically ill patients. It allows recognition that the patient is in shock and characterization of the type of circulatory failure. This step is crucial to initiate supportive interventions and to cure the cause responsible for the development of shock. Evaluation of tissue perfusion allows identification of the patient insufficiently resuscitated and also to trigger therapeutic interventions. Monitoring tissue perfusion can be achieved by lactate, venoarterial gradients in PCO_2_, and central venous or mixed venous oxygen saturation. Ultimately, monitoring the microcirculation may help not only to identify alterations in tissue perfusion but also to identify the type of alterations: diffuse decrease in microvascular perfusion versus heterogeneity in the alterations, as in sepsis, with well perfused areas in close vicinity to poorly perfused areas. Regarding supportive therapy, a step-by-step approach is suggested, with fluid optimization followed by vasoactive support to preserve perfusion pressure and global and regional blood flows. The different variables should be integrated into decision and management pathways, and therapies adapted accordingly.

## Background

Evaluation of the cardiovascular profile of critically ill patients is one of the most common explorations performed in the ICU. Several tools can be used to evaluate the hemodynamic state of a patient but the interest of a given technique goes well beyond its invasiveness. Even though ideally less invasive methods should be preferred over more invasive methods, the reliability of cardiac output measurements with some of the noninvasive techniques is sometimes questioned in patients in shock states [1]. More importantly, the interest in hemodynamic monitoring in shock states goes beyond the simple measurement of cardiac output and the interest in the multiple derived variables often orients the choice for one technique over another.

In patients with shock, the decision to use a given hemodynamic technique should be based on what the physician expects from the measured variables. The following four important questions should be addressed: Is the patient in shock? What is the type of shock? Is tissue perfusion adequate, and if not how to improve it? Is cardiovascular function adequate?

## Recognition of shock

In a recent consensus, shock was defined as “a life-threatening, generalized form of acute circulatory failure associated with inadequate oxygen utilization by the cells”. Shock is thus a state in which the circulation is unable to deliver sufficient oxygen to meet the demands of the tissues, resulting in cellular dysoxia. Hence, VO_2_ by the tissues becomes limited by DO_2_. In the past, VO_2_/DO_2_ relationships were evaluated at the bedside, but this was quite cumbersome and subject to errors in measurements. Accordingly, surrogate markers are often used to identify shock, among which are measurements of blood lactate levels as lactate levels rise sharply when DO_2_ reaches the point at which VO_2_ becomes dependent on DO_2_. Shock is also associated with signs of impairment of tissue perfusion (skin vasoconstriction or mottling, acrocyanosis, impaired capillary refill time, impaired microcirculation, increased venoarterial PCO_2_ gradient), but these may already be present before the onset of VO_2_/DO_2_ dependency.

Importantly, even though hypotension is often encountered in shock states, shock may sometimes develop without hypotension (especially in previously hypertensive patients). Accordingly, a patient who presents signs of impaired tissue perfusion and increased plasma lactate levels should be considered a patient in shock.

## What is the type of shock?

**Fig. 1:**
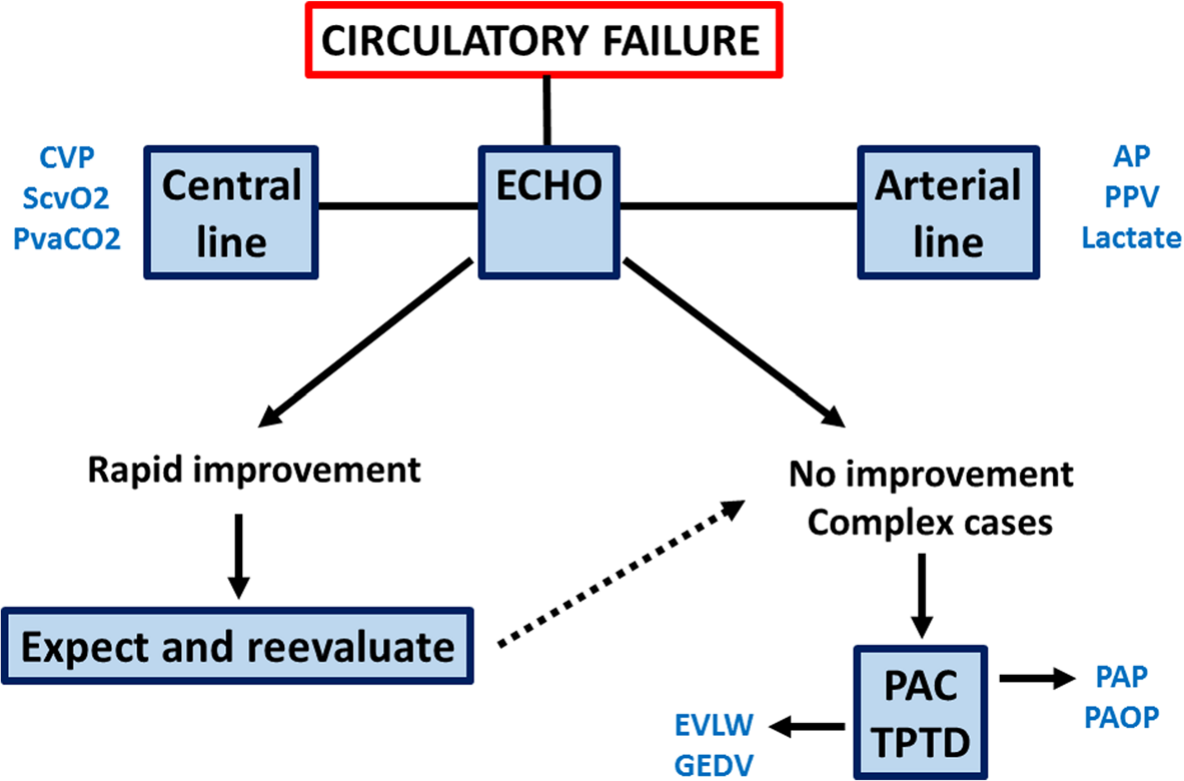
Suggested evaluation and monitoring strategy for hemodynamic assessment of a patient with circulatory failure. PAC pulmonary artery catheter, TPTD transpulmonary thermodilution, PAP pulmonary artery pressure, PAOP pulmonary artery occluded pressure, EVLW extravascular lung water index, GEDV global end diastolic volume, CVP central venous pressure, ScvO_2_ central venous oxygen saturation, PvaCO_2_ venoarterial difference in PCO_2_, AP arterial pressure, PPV pulse pressure variations

The next important point would be to evaluate the type of shock that the patient presents, as it would orient not only supportive therapies but also causal management. There are four types of shock: hypovolemic, cardiogenic, obstructive, and distributive. Several hemodynamic tools can be used to determine the type of shock. However, echocardiography is the most convenient tool as it can rapidly lead to the diagnosis of the type of shock. In a study including 108 patients in shock, echocardiography diagnosed the type of shock within 4.9 ± 1.3 min [2]. There was also an excellent agreement between the various observers. Accordingly, echocardiography is now recognized as the preferred initial modality to evaluate the type of shock [3]. Accordingly, many patients can be monitored just with an arterial line and a central line, in addition to initial echocardiography (Fig. 1). When the patient does not respond to initial therapy, or in complex cases, additional hemodynamic monitoring is recommended. In these cases, the pulmonary artery catheter or transpulmonary thermodilution are the preferred methods [3].

**Fig. 2:**
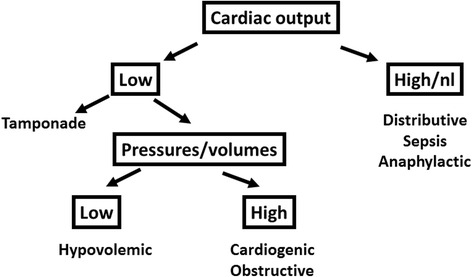
Diagnostic algorithm for the characterization of different types of shock. nl normal

The advantage of the pulmonary artery catheter and transpulmonary thermodilution over other less invasive techniques is that these provide not only reliable cardiac output measurements even in extreme conditions, but also additional variables that help to understand the cardiovascular profile. The pulmonary artery catheter allows the measurement of intravascular pressures while transpulmonary thermodilution estimates intravascular volumes. With the measurements of intravascular pressures and volumes it is feasible to identify the type of shock (Fig. 2).

## Evaluation and therapeutic approach of tissue hypoperfusion

### Lactate

Measurements of blood lactate levels can be useful to detect occult tissue hypoxia and also to monitor the effects of therapy.

Lactate is a byproduct of glycolysis. In the first phase, which is anaerobic and occurs in the cytoplasm, glucose is transformed into pyruvate. If oxygen is present, the pyruvate enters the mitochondria to participate in the second phase of reactions, generating ATP, H_2_O, and CO_2_, but can also be transformed into lactate if oxygen is lacking or in some cells that do not contain mitochondria. In normal conditions, most pyruvate enters the mitochondria, so that the normal lactate/pyruvate ratio is around 10. In anaerobic conditions, pyruvate cannot enter the mitochondria and massive amounts of lactate will be produced and the lactate/pyruvate ratio increases well above 20.

It is commonly accepted that hyperlactatemia is mostly of hypoxic origin in critically ill patients with circulatory failure. However, tissue hypoxia cannot be sustained for long periods of time without inducing cell death, as the energy produced by anaerobic metabolism is quite low compared to aerobic metabolism. Mild hyperlactatemia in hemodynamically stable septic patients is often not related to tissue hypoxia. Sepsis-induced inflammatory mediators accelerate aerobic glycolysis, increasing pyruvate availability. This increase in pyruvate availability can lead to increased lactate production, even in the presence of large amounts of oxygen. In addition to the increased lactate production, a decrease in lactate clearance can participate in hyperlactatemia. In patients with shock, hyperlactatemia is often of hypoxic origin shortly after admission, while hyperlactatemia persisting for more than 1 day is often of nonhypoxic origin [4].

### Venoarterial PCO_2_ gradients

According to the Fick equation, the difference between venous and arterial PCO_2_ is inversely related to flow, provided CO_2_ production remains constant. The normal PvaCO_2_ gradient is lower than or equal to 6 mmHg. When ScvO_2_ is abnormal, the increase in PvaCO_2_ mostly reflects the decrease in cardiac output. When ScvO_2_ is normal, an increase in PvaCO_2_ reflects microcirculatory dysfunction [5, 6].

Interestingly, the increase in PvaCO_2_ can also reflect occurrence of anaerobic metabolism. In anaerobic conditions, aerobic CO_2_ production decreases but CO_2_ is also generated by buffering the protons generated by ATP hydrolysis so that CO_2_ production becomes higher than VO_2_. Accordingly, the respiratory quotient becomes higher than 1. The respiratory quotient can be approximated by dividing PvaCO_2_ by arteriovenous O_2_ difference, and a ratio above 1.3 suggests anaerobic metabolism. In order to eliminate potential interference with the Haldane effect, the ratio between venoarterial CO_2_ content/arteriovenous O_2_ difference is computed, with a ratio above 1 reflecting anaerobic metabolism.

In patients with septic shock, Ospina-Tascon et al. [7] demonstrated that a venoarterial CO_2_ content/arteriovenous O_2_ difference ratio above 1 was associated with a poor outcome.

### How to combine lactate, venoarterial PCO_2_ gradients, and ScvO_2_ measurements?

**Fig. 3:**
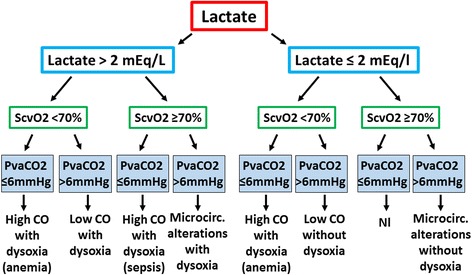
Interpretation of measurements of lactate, central venous oxygen saturation, and venoarterial PCO_2_ gradients. PvaCO_2_ venoarterial difference in PCO_2_, ScvO_2_ central venous oxygen saturation, Microcirc. microcirculation, Nl normal

Combining lactate, CO_2_ differences, and ScvO_2_ can help to discriminate between normal and abnormal patterns, and to identify which part of the system is mostly contributing to these alterations. Of course, combination of several processes may occur (i.e., low cardiac output and microvascular alterations) but one often prevails over the other. The decision algorithm is shown in Fig. 3.

### Microcirculation assessment

In patients with circulatory failure, organ perfusion is often decreased as a result of a low cardiac output or perfusion pressure. However, tissue perfusion can remain altered even after achievement of within-target cardiac output and arterial pressure. The microcirculation is the part of the circulation responsible for fine tuning the distribution of flow at the organ level. Alterations in microvascular perfusion occur in sepsis and septic shock [8], as well as in cardiogenic shock [9]. The severity and the duration of microcirculatory alterations are related to the occurrence of organ dysfunction and risk of death [10]. Different mechanisms have been implicated in the development of these alterations including loss of communication between vascular segments, impaired endothelial vasoreactivity, alterations in red and white blood cell rheology, alteration in endothelial glycocalyx, platelet aggregation, and microthrombosis. In addition to the alterations in microvascular perfusion, alteration in microvascular endothelium is associated with activation of coagulation and inflammation, reactive oxygen species generation, and permeability alterations [11].

Microvascular perfusion can be monitored by several techniques, but direct videomicroscopy is probably the most appropriate method as it allows one to detect heterogeneity of perfusion, which is the hallmark of these alterations [12]. In normal conditions, microvascular perfusion is quite homogeneous with a density of perfused vessels that increases or decreases in proportion to metabolic needs. In septic shock, microvascular perfusion is characterized not only by a decrease in vessel density but mostly by heterogeneity in perfusion, with nonperfused vessels in close vicinity to well perfused vessels [8–10]. The consequence is a decrease in perfused vascular density and microvascular shunting, resulting in hypoxic zones while venous saturation is increased.

## Therapeutic approach

The therapeutic approach should be guided by the hemodynamic monitoring variables. An important question is whether therapy should be protocolized or individualized. Protocolized hemodynamic resuscitation is based on the concept that similar target values should be achieved in all patients, and these targets are determined on the basis that the majority of the patients reaching these goals would have a better outcome. However, some patients may be exposed to the side effects of the therapies applied to reach these goals when reaching lower goals was sufficient.

## Fluid management

Fluid management is the cornerstone for the resuscitation of the septic patient, aiming at improved tissue perfusion through an increase in cardiac output. While most patients are usually fluid responsive in the initial stages, fluid resuscitation becomes more challenging at a later stage as many patients may no longer be fluid responsive. In addition, a positive fluid balance, especially at later stages, is associated with poor outcome. Hence, several approaches can be used to predict the response to fluids. Static measurements of preload such as CVP are only useful at their extreme values [13]. Targeting a specific CVP value is only valid at the population level, as close to two-thirds of the patients respond to fluids when baseline CVP is below 8 mmHg and two-thirds do not respond when CVP is higher than 12 mmHg [14]. Use of dynamic tests allows prediction of the response to fluids at the individual level. Among the most attractive tests, respiratory variations in stroke volume (directly measured by different pulse contour techniques or by Doppler ultrasounds) or its derivative variations in pulse pressure can reliably predict the response to fluids when several pre-requirements are met (absence of arrhythmias, tidal volume larger than 8 ml/kg, no respiratory movements, etc.). Passive leg raising is another reliable test, but this requires a fast-response cardiac output monitor and can be fastidious if it has to be repeated frequently. These dynamic tests are now recommended in recent guidelines [3].

**Fig. 4:**
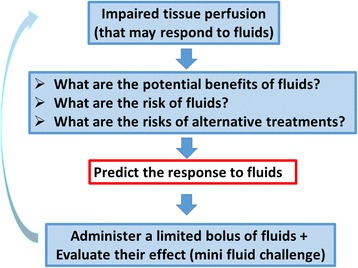
Decision tree for the administration of fluids

An alternative is the so-called mini fluid challenge. It has been proposed that administration of a limited amount of fluids (~100 ml) in a short period of time may predict the administration of further fluids [15]. This concept is only partly valid: if a patient does not respond to the first bolus, there is very limited chance that they will respond to further fluid administration. However, a positive response to the first bolus does not imply a response to further fluid administration (and the trials were biased as the response to the first bolus was integrated in the assessment of the total amount of fluids). If anything it should be the contrary, as the likelihood of the response to fluids in preload responsive patients (and thus on the ascending part of the Starling relationship) is higher with the initial bolus than with later boluses. It is nevertheless safer to repeatedly administer small boluses of fluids, evaluating their effects, and to predict the response to fluids before the next bolus, than to administer large boluses of fluids once patients are predicted to be fluid responsive. The decision algorithm is shown in Fig. 4.

### Blood pressure

Blood pressure is a resuscitative target that has been investigated broadly. Guidelines recommend reaching a mean arterial pressure of 65 mmHg while recognizing that some patients may require higher values [3, 16]. This target is based on observational data reporting higher death rates when this target is not reached, while reaching higher values was not associated with a better outcome. Nevertheless, small-sized studies have shown a huge variability in the response to increasing mean arterial pressure to higher targets, suggesting that some patients may benefit from higher values [17, 18]. In a multicentric randomized trial including 776 patients in septic shock, no difference in 28-day mortality was observed between patients allocated to 65 or 85 mmHg. Interestingly, there was a lower incidence of acute kidney injury in the previously hypertensive patients allocated to the higher target [19]. As a result of the higher doses of norepinephrine that were required to reach the higher target, the incidence of atrial fibrillation was also significantly higher in that group. Hence, higher targets cannot be recommended in all patients. This study nicely illustrates that minimal targets should be reached in all patients and that, if needed, higher targets can be considered in some patients. If higher targets are considered, it is important to evaluate whether the patient responds adequately to the therapy, illustrating the need to measure the variable that is expected to be corrected.

### Early goal directed therapy vs individualized approach

Early goal directed therapy (EGDT) is the second target that has been studied extensively in septic shock patients. Of note, the term EGDT has become evasive as it was interpreted in many directions, so its initial meaning is sometimes lost. For some, EGDT represents aggressive fluid resuscitation, sometimes based on CVP, for others it represents optimal early hemodynamic resuscitation, for others the prompt use of broad-spectrum antibiotics, and so forth. EGDT consists of the optimization of oxygen transport (DO_2_) based on measurements of ScvO_2_ and administration of fluids, red blood cell transfusions, and inotropes. The concept of EGDT was initially tested by Rivers et al. [20] who demonstrated in a randomized trial including 263 patients with septic shock that EGDT markedly decreased 28-day mortality from 49% in the control group to 33% in EGDT patients. Even though the results of this study created an inspiring wave for early resuscitation, they also generated some debate, especially as the concept was brought into a resuscitation package, the so-called bundles, that initially were suggested as a help to guide resuscitation of septic patients, especially in difficult environments (when critical care specialists are not available), and were then moved into a law-enforced mandatory bundle. This was of course highly criticized as some part of the bundles (i.e., CVP/MAP) used elements that were present in both trial arms and could not be responsible for the differences in outcome between the two arms.

Several large-scale randomized trials failed to reproduce these results [21]. Does this mean that the concept is dead? Probably not, as many factors differed between the different trials [22]. First, ScvO_2_ at inclusion was already within target in more than 75% of the cases in the recent trials, while it was markedly abnormal in the Rivers et al. trial. Second, the patients included in the recent trials were much less severe, as reflected by their mortality rates but also by the fact that up to 20% of the patients were not admitted to the ICU, even though presenting the same inclusion criteria at baseline. As there was no harm objectivized in the new trials, a reasonable approach may be that EGDT should not be used in all patients with sepsis but can (should?) still be implemented in the most severe, especially if presenting with low ScvO_2_ at baseline. It should nevertheless be noted that high ScvO_2_, together with signs of tissue hypoperfusion and organ dysfunction, is not reassuring. High ScvO_2_ is associated also with a poor outcome and may represent microvascular alterations as well as mitochondrial dysfunction.

The second aspect of the bundle that was criticized was the use of CVP to guide fluid resuscitation. Indeed, initial bundles recommended maintaining CVP between 8 and 12 mmHg. While this may be relatively satisfactory on a statistical basis, as many (2/3) of the patients with CVP values below 8 mmHg would respond to fluids and as most of the patients with CVP > 12 mmHg will not respond to fluids [23], the use of CVP for the prediction of response to fluids is far from optimal even though used frequently [24]. The new version of the Surviving Sepsis Campaign Guidelines takes this aspect into account for the guidance of fluid resuscitation: “We recommend that, following initial fluid resuscitation, additional fluids be guided by frequent reassessment of hemodynamic status (Best Practice Statement)” [16]. The frequent reassessment is based on the use of clinically relevant variables, hemodynamic monitoring, and the use of dynamic over static variables to predict fluid responsiveness, where available [16]. This is a major advance toward individualization of EGDT procedures that is a further step forward in improving the care of septic patients [25].

### Treatment of microvascular disorders

How to manipulate the microcirculation? Increasing flow without recruiting the microcirculation is ineffective. Fluids, at the early stages, improve the microcirculation but this effect is blunted at later stages [26, 27]. Interestingly, when fluids had positive effects on the microcirculation, this translated into an improvement in organ dysfunction score the next day [28]. Dobutamine may increase microvascular perfusion, but this effect is often limited [29, 30]. The use of vasodilatory agents has been proposed [31] but there is still insufficient evidence to support their use [32]. In particular, due to their absence of selectivity, these can also dilate already perfused vessels and lead to a steal phenomenon. Modulation of endothelial nitric oxide synthase appears promising [33]. More data are required before using the microcirculation as a direct therapeutic target, it is nevertheless important to understand what could be the potential impact of our interventions on the microcirculation.

## Evaluation of and therapeutic approach for cardiovascular dysfunction

Cardiac output measurements provide only one part of the information. However, it is very important to evaluate whether or not cardiac output is adequate. Adequacy of cardiac output can be evaluated by ScvO_2_ or SvO_2_, in addition to signs of tissue perfusion.

In addition, measurements of filling pressures or volumes of cardiac chambers can be helpful to evaluate the cardiovascular performance [1].

When deciding to treat or not an alteration in myocardial contractility, it is important to evaluate the consequences of the impaired contractility: is cardiac output inadequate and is it associated with impaired tissue perfusion? Indeed, the relationship between contractility and cardiac output is relatively loose [34]. Some patients may have decreased contractility with preserved cardiac output, and these patients should not be treated with inotropic agents [34]. Other patients may have a low cardiac output and these patients should also not be treated with inotropic agents. Only patients with a low cardiac output related to an impaired contractility may benefit from inotropic agents.

A recent trial illustrated the need for individualizing therapy in this domain. In the trial administering levosimendan to patients with septic shock, the addition of levosimendan to standard treatment was not associated with a lower incidence of sepsis-induced organ dysfunction or lower mortality [35]. However, levosimendan was associated with higher risk of tachyarrhythmia. Admittedly, this trial was not optimally designed, as cardiac output and cardiac function were not evaluated so that patients with high cardiac output and/or high contractility may have received levosimendan even when not required or even when contraindicated. Indeed, one-fifth of the patients with septic shock may present left ventricular outflow tract or mid-ventricular obstruction [36], which contraindicates the use of inotropic stimulation. Hence, individualization of therapies, based on hemodynamic assessment, is the preferred approach in these patients.

## Conclusion

Hemodynamic assessment remains an important aspect of the care of the critically ill patient. Several tools are available, and the selection of the different tools should be based on the potential interest in a given patient of the measured variables. More than the tools, the use of the measured variables is of critical relevance. The different variables should be integrated into decision and management pathways, and therapies adapted accordingly.

## Abbreviations

CVP: Central venous pressure
DO_2_: Oxygen delivery
EGDT: Early goal directed therapy
ScvO_2_: Central venous oxygen saturation
VO_2_: Oxygen consumption
